# Radio wave attenuation by a large-scale photonic crystal sculpture

**DOI:** 10.1038/s41598-025-95986-9

**Published:** 2025-04-10

**Authors:** David Röhlig, Vincent Laude, Ralf Zichner, Felix Thieme, Angela Thränhardt, Thomas Blaudeck

**Affiliations:** 1 Institute of Physics, Chemnitz University of Technology, Reichenhainer Straße 70, 09126 Chemnitz, Germany; 2 Université Marie et Louis Pasteur, CNRS, FEMTO-ST Institute, 15B avenue des Montboucons, 25030 Besançon, France; 3 Faculty of Electrical Engineering and Information Technology, Chemnitz University of Technology, Reichenhainer Straße 70, 09126 Chemnitz, Germany; 4 Fraunhofer Institute for Electronic Nano Systems (ENAS), Technologie-Campus 3, 09126 Chemnitz, Germany; 5 Research Center for Materials, Architectures and Integration of Nanomembranes (MAIN), Chemnitz University of Technology, Rosenbergstraße 6, 09126 Chemnitz, Germany

**Keywords:** Photonic crystal, Electromagnetic crystal, Band gap, FEM, Radio waves, Mobile communication, Photonic devices, Applied physics, Optical physics

## Abstract

In this work, we present an artwork that embodies the by far largest photonic crystal ever published, operating at radio frequencies. We demonstrate both theoretically and experimentally the existence of band gaps. While photonic crystals are typically investigated on the nano and micro scale, our aim is to present not only a remarkable example of art with scientific relevance but also to highlight the potential for large-scale applications that have so far been underrated.

## Introduction

The splendor of a butterfly’s wing, illuminated by the sun, unveils not only aesthetic magnificence but also the conceptual beauty of the underlying mechanism: the intricate chitin nanostructure of its scales interacts with light^1–3^, blurring the lines between science and art. These structures, if periodic, are commonly recognized as photonic crystals, first described in 1987 both by Yablonovitch^4^ and John^5^, playing pivotal roles not only in nature but also in diverse technological applications^6^. They consist of different dielectric materials or even metals^7,8^ arranged in a periodicity comparable to the wavelength of light.

Remarkably, these crystals are predominantly examined at the meso- or microscopic level, possibly due to human fascination for visible light, yet their potential applications extend beyond this scope. The situation is different in the realm of acoustics: here, the concept of phononic crystals has been coined as analogous to photonic crystals^9,10^. In particular, a publication exists for a comparable art installation made of a two-dimensional periodic structure of cylinders made from stainless steel, showcasing the formation of an acoustic band gap for the propagation of sound waves and hence the observation of a frequency-dependent sound attenuation^11^.

For electromagnetic waves, it is uncommon to consider the concept of periodicity at such large scales. Around the turn of the millennium, there was a strong interest in photonic structures operating at radio frequencies, as emphasized by Yablonovitch and others^12,13^. Surprisingly, there still appears to be no literature addressing photonic crystals, or in this context sometimes referred to as electromagnetic crystals^12–14^, with lattice constants exceeding a few centimeters. They have been studied in the millimeter to centimeter range for one-dimensional multilayer structures^15–17^, as well as for two-^18^ and three-dimensional structures^19–21^, operating primarily in the radio wave regime.

**Fig. 1:**
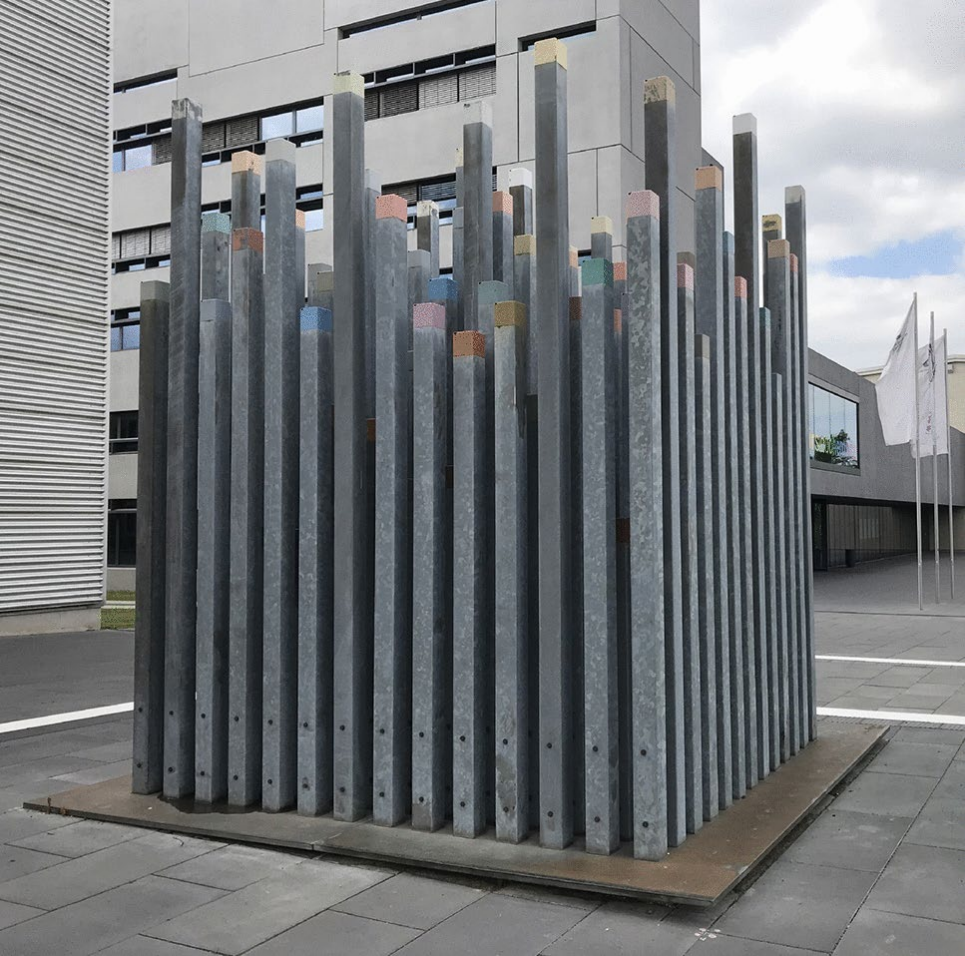
Artwork “Denk- und Wahrnehmungsmodell zum Phänomen der Farbe” by Stefan Nestler from 1998, consisting of 187 colored steel stelae.

In this article, we present an investigation of an unusual example of a two-dimensional metallic photonic crystal that far exceeds the scale of previously reported structures. Its periodicity is relevant to radio wave frequencies pertinent to network standards such as 4 or 5G^22^. The structure under study is the artwork “Denk- und Wahrnehmungsmodell zum Phänomen der Farbe” (translated as “Thinking and Perception Model on the Phenomenon of Colour”) from 1998 created by artist Stefan Nestler and located in front of the central lecture hall of Chemnitz University of Technology. This piece of art consists of 187 periodically arranged hollow steel columns, standing upright and in parallel. Interestingly, configurations involving rectangular cavities within a metallic waveguide are well-known to function as bandpass filters^23,24^. More notably, the two-dimensional structure of the stelae formally satisfies the criteria of a finite photonic crystal and therefore joins the ranks of art with scientific relevance. Our objective is to adopt a cross-scale perspective on photonic crystals, further unraveling their potential and application possibilities.

## Modeling the structure

**Fig. 2:**
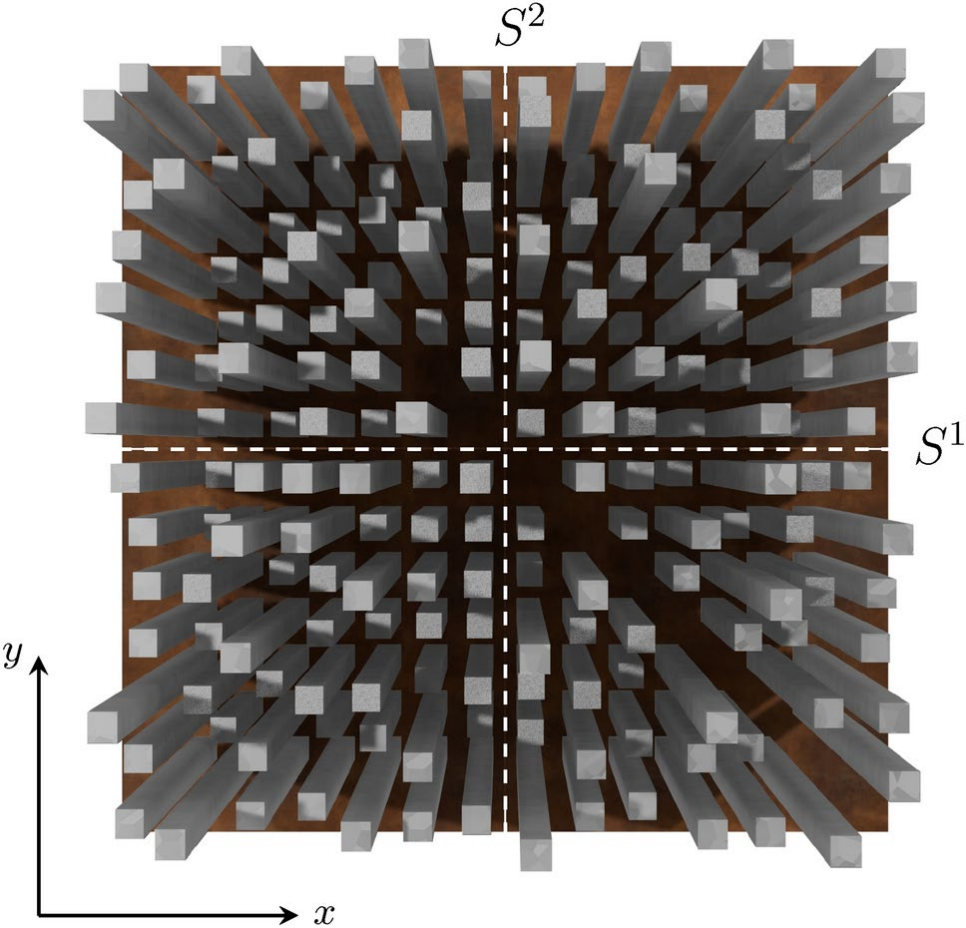
Visualization of the sculpture (generated with Blender ^25^), viewed from above, consisting of a $14\times 14$ grid of steel stelae, with nine missing elements along one of the primary diagonals. Two measurement scenarios are indicated by dashed lines, corresponding to $S^1$ and $S^2$.

The stelae are arranged on a $4 \times 4$ square meter metal plate, forming a $14 \times 14$ array with a lattice constant of 0.24 meters (see Fig. 1). Along one of the main diagonals, nine columns are missing, as illustrated in Fig. 2. The measured heights of the stelae range from 0.99 to 3.97 meters, each with a base area of $120\times 120$ millimeters. They are hollow, and anchored in metal rails. Exposure to local weather conditions has resulted in minor surface inhomogeneities. The overall wall thickness is approximately 5 millimeters.

For describing wave phenomena within a photonic crystal, we begin with an eigenvalue equation, which can be directly derived from Maxwells equations by assuming time harmonic field solutions $\varvec{F}(\varvec{r},t) =\varvec{F}(\varvec{r})\, e^{i\omega t}$. In two dimensions, the governing equation can be decoupled for two distinct modes, yielding:

1 $$\begin{aligned} -\nabla \cdot \Bigl (a(\varvec{r})\, \nabla F_z(\varvec{r})\Bigr ) = b(\varvec{r})\, \frac{\omega ^2}{c^2} F_z(\varvec{r}), \end{aligned}$$

where $\omega$ is the angular frequency and *c* represents the speed of light in vacuum. This formulation applies to both TM modes, where the magnetic field is oriented along the *z* direction (perpendicular to the plane of periodicity), and TE modes, where the electric field is out of plane. Depending on the mode, the parameters $a(\varvec{r})$ and $b(\varvec{r})$ are defined to be:

$$\begin{aligned} \begin{array}{c|c c c} \text {mode} & \; F_z(\varvec{r})\;& \; a(\varvec{r})\; & \; b(\varvec{r})\; \\ \hline \text {TM} & H_z(\varvec{r})& \;\epsilon (\varvec{r})^{-1} & 1 \\ \text {TE} & E_z(\varvec{r})& 1 & \epsilon (\varvec{r}) \\ \end{array} \end{aligned}$$

### Band structure calculations

For calculating the band structures, we employed the finite element method (FEM), which necessitates a variational formulation. To derive it, we begin by multiplying the eigenvalue Eq. (1) with a test function $\tilde{F_z}$. Next, we integrate over the domains $\Omega$ within the unit cell and apply the divergence theorem. Given that, in the context of band structures, we are exclusively concerned with the propagation of Bloch waves within an infinite periodic structure, we incorporate Bloch wave solutions $F_z(\varvec{r},t)=F_z(\varvec{r}) \exp (i\omega t-i\varvec{k}\cdot \varvec{r})$ for both the field component and the test function, so that we derive the following expression:

2 $$\begin{aligned} 0&= \frac{\omega ^2}{c^2} \int _{\Omega } \tilde{F}_z \, b\, F_z \, \textrm{d}\varvec{r}\nonumber \\&- \int _{\Omega } (\nabla + i\varvec{k}) \tilde{F_z} \cdot \left[ a \, (\nabla - i\varvec{k}) F_z \right] \, \textrm{d}\varvec{r} .\nonumber \\&+ \int _{\partial \Omega _{i}} \tilde{F_z} \left[ a \, (\nabla - i\varvec{k}) F_z \right] \cdot \varvec{n} \; \textrm{d}s \quad \forall \tilde{F_z}. \end{aligned}$$

Functions $\tilde{F_z}$ and $F_z$ in this equation are understood as the periodic part of Bloch waves. We could, in principle, decompose the integrals over the distinct subregions $\Omega$ of the air matrix and the inclusion; however, as we shall see shortly in the next section, it suffices to mesh only the matrix. The final term represents integrals along the internal boundaries $\partial \Omega _i$, incorporating the normal vectors $\varvec{n}$. At the external boundaries $\partial \Omega _e$, periodic boundary conditions are applied and lead to the cancelling of boundary terms along $\partial \Omega _e$.

### Boundary conditions

**Fig. 3:**
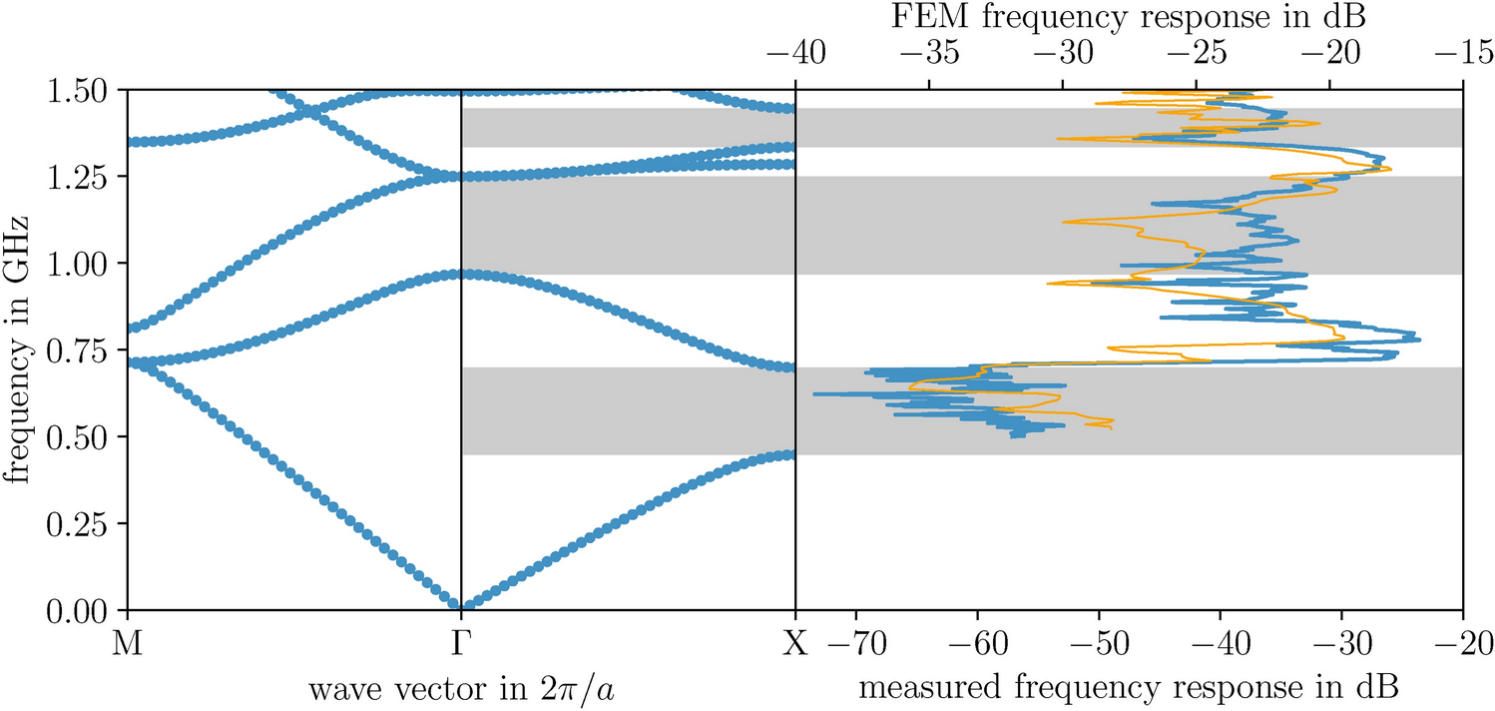
Theoretical band structure predictions for TM polarization, obtained using FEM on a triangular mesh of P2 elements with a resolution of 25 nodes per lattice constant at both the external and internal boundaries, are compared both to the simulated and measured frequency response function of the stele artwork. The simulation cell, depicted in Fig. 5, features the same resolution as that used for the band structure calculation. Measurements were taken along the $\Gamma$-X path in reciprocal space under TM polarization, wherein the magnetic field is oriented perpendicular to the plane of periodicity. The spectra fall within a frequency range of $0.5-1.5$ GHz.

Although the scatterer material exhibits dispersion, we neglect the explicit frequency dependence by applying metallic boundary conditions, which, as will be demonstrated later, show good agreement with experimental results. Specifically, we assume an infinitely high conductivity ($\sigma \rightarrow \infty$) within the metal, such that the electric field vanishes at the material boundary $\partial \Omega _i$ ($\varvec{E}\rightarrow 0$). This is heuristically supported by Ohm’s law, $\varvec{j}=\sigma \varvec{E}$, for a finite current density $\varvec{j}$. Given that the tangential component of the electric field must remain continuous across the material interface $\partial \Omega _i$, the condition $\varvec{E}_1 \times \varvec{n} = \varvec{E}_2 \times \varvec{n}$, where $\varvec{n}$ denotes the normal vector, must hold. Assuming the second region behaves as a so called perfect electric conductor (PEC), the field $\varvec{E}_2$ is zero, resulting in the following condition at the boundary $\partial \Omega _i$:

3 $$\begin{aligned} \varvec{E}_1 \times \varvec{n} = 0. \end{aligned}$$

This considerably simplifies the problem. For the calculations, we can now treat the hollow steel inclusions as filled, since the fields can not enter the PEC. Moreover, there is no need to mesh its domain within the unit cell, as it can be entirely described by the boundary integral. Given that the matrix material is air, we set $\epsilon =1$, which leads to $a=b=1$ in the weak formulation, as shown in Eq. (2). Consequently, the weak form is identical for both mode types, except for the application of boundary conditions. For TE modes, we simply apply Dirichlet boundary conditions, forcing $F_z = E_z = 0$ along the boundary. For TM modes, we must consider Ampère’s law in the time-harmonic regime, $\nabla \times \varvec{H} = (\sigma - i\omega \epsilon )\varvec{E}$. By inserting it into the PEC condition Eq. (3), we obtain:

4 $$\begin{aligned} 0 = \varvec{n} \cdot (\nabla - i\varvec{k}) H_z . \end{aligned}$$

In the weak form Eq. (2), this is a Neumann boundary condition. The boundary integral in Eq. (2) then naturally vanishes.

**Fig. 4:**
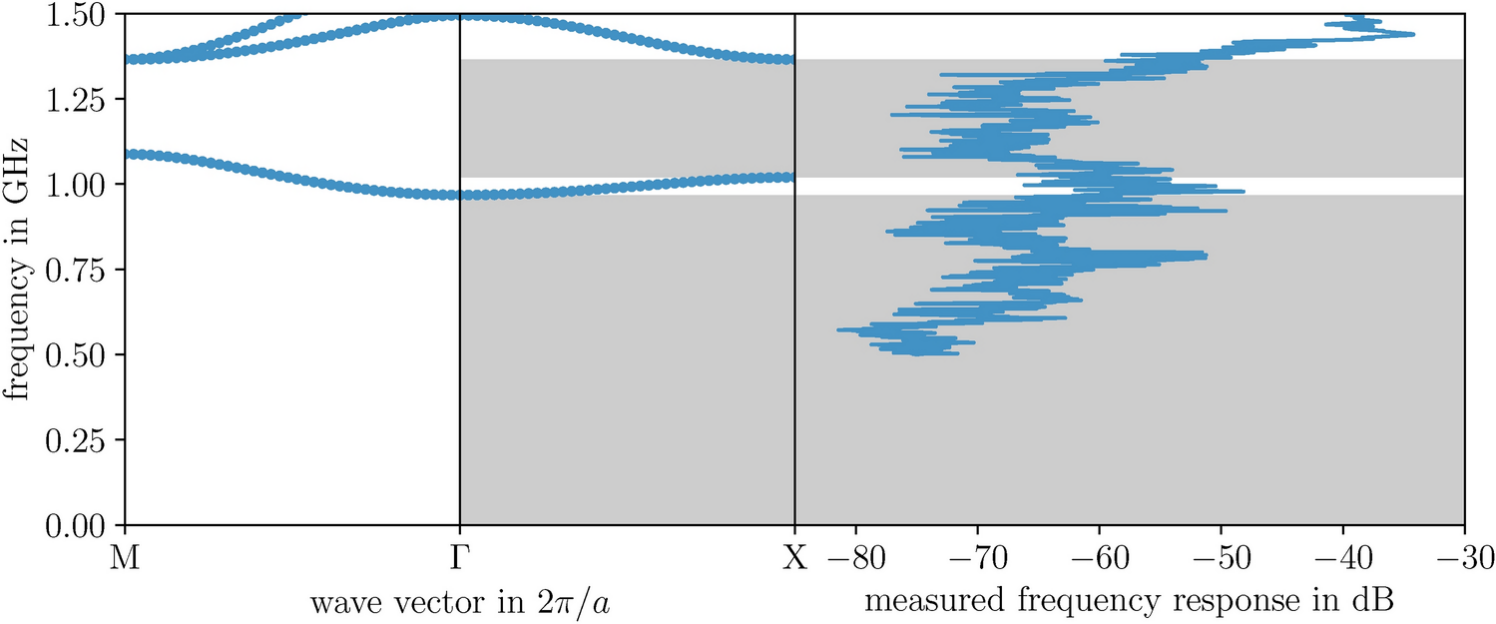
Theoretical band structure predictions are compared with the measured and simulated frequency response function, analogous to Fig. 3, but for TE polarization, where the electric field is oriented perpendicular to the plane of periodicity.

### Transmission properties

**Fig. 5:**
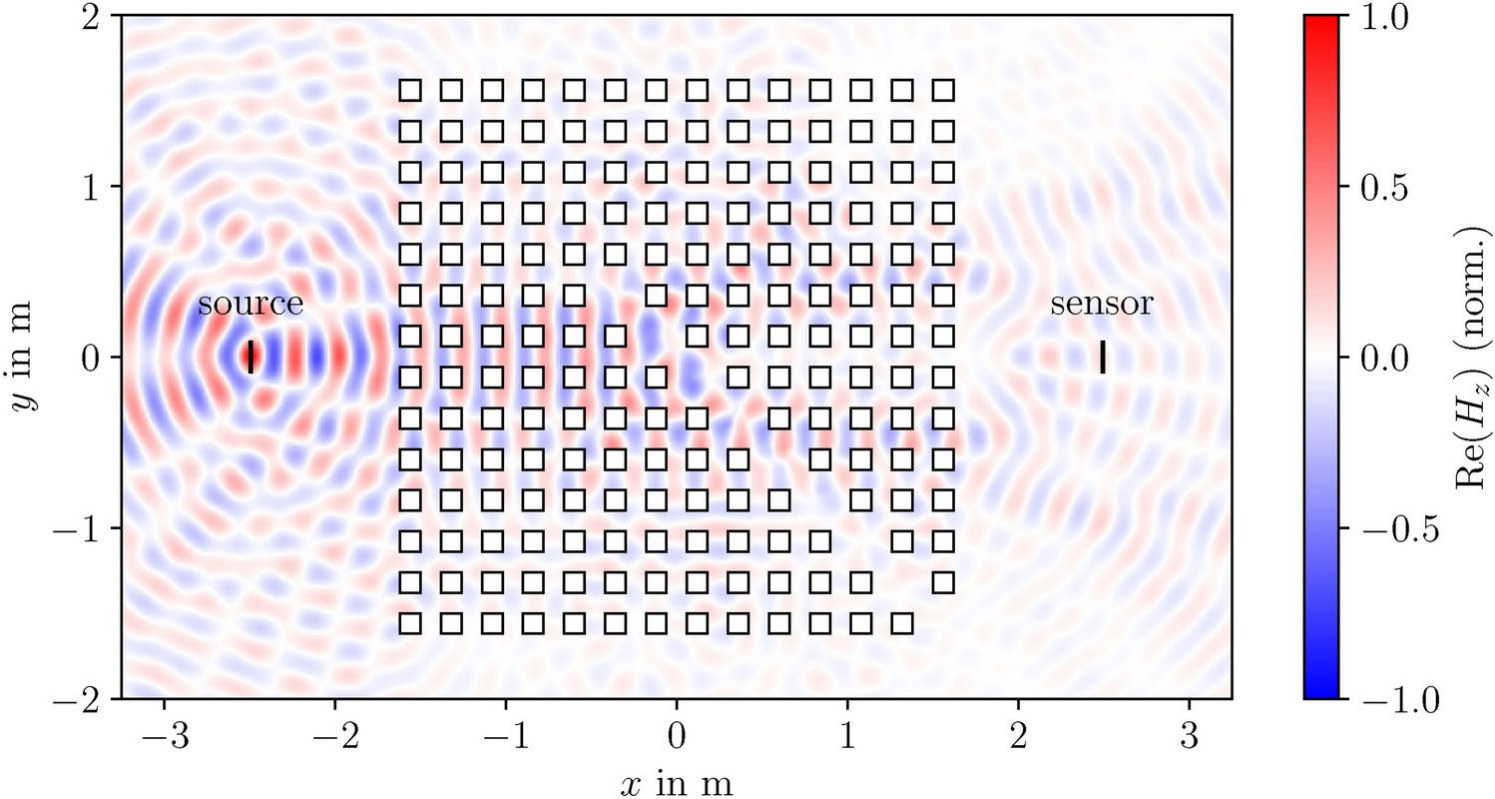
The normalized field distribution for TM modes at a frequency of 1.25 GHz was computed using the FEM with P2 elements with a resolution of 25 nodes per lattice constant, by applying radiation boundary conditions in both the *x*- and *y*-directions. This simulation cell serves as the basis for calculating the frequency response curves shown in Fig. 3 and 4. The source is defined on the left, while the frequency response is evaluated on the right, corresponding to the measurement scenario $S_{21}^1$.

To investigate the transmission properties of the artwork, we return to the variational formulation (2). Since – unlike for band structures – we are only concerned with a finite structure, we adhere to the previous derivation but $F_z$ now represents the electric or magnetic field and not a Bloch wave. By prescribing a specific value on a defined line within the simulation cell, as indicated in Fig. 5, we can introduce a source. In this case, the left-hand side of the equation is no longer zero. Since for a plane wave, $\nabla F_z = -i\varvec{k}_0\, F_z$, we obtain

5 $$\begin{aligned} \tilde{F_z} \bigl ( a\nabla F_z \bigr ) \cdot \varvec{n} = -\tilde{F_z}\, a F_z\, i\varvec{k}_0\cdot \varvec{n} \end{aligned}$$

for the expressions inside the boundary integrals. To enable plane waves to freely exit the simulation cell, we impose radiation boundary conditions at the external cell borders $\partial \Omega _e$ in the *x* and *y* directions, putting $\varvec{k}_0\cdot \varvec{n} = \omega /c$. Consequently, we arrive at:

6 $$\begin{aligned} \int \limits _\text {source}\tilde{F_z}\, F_0\, \frac{i a\omega }{c} \; \textrm{d}s&= -\frac{\omega ^2}{c^2} \int _{\Omega } \tilde{F}_z \, b\, F_z \, \textrm{d}\varvec{r}\nonumber \\&+ \int _{\Omega } \nabla \tilde{F_z} \cdot \left( a \, \nabla F_z \right) \, \textrm{d}\varvec{r} \nonumber \\&+ \int _{\partial \Omega _e}\tilde{F_z}\, F_z\, \frac{i a\omega }{c} \; \textrm{d}s \quad \forall \tilde{F_z}. \end{aligned}$$

Note that Dirichlet (for TE waves) or Neumann (for TM waves) boundary conditions are still imposed along the boundaries $\partial \Omega _i$ defining the inclusions. Here, $F_0$ denotes the initial field generated by the source. To ultimately determine the frequency response, we integrate along the line of the sensor position, as depicted in Fig. 5:

7 $$\begin{aligned} T = \frac{1}{ F_0 l} \int \limits _\text {sensor} F_z\; \textrm{d}s . \end{aligned}$$

Both the sensor and the source have a length *l*, corresponding to a dipole length in the experiment. For the measurements, we employ logarithmic antennas composed of numerous dipoles of varying sizes and distances (from the antenna base) – each responsible for different frequency ranges. In the simulation, these geometric aspects were taken into account. Consequently, depending on the frequency, both the position and size of the sensor regions vary according to the geometry of our antenna model. Since, for TE modes, the dipoles are oriented out of the plane of periodicity, in a two-dimensional simulation, this can only be considered for TM modes. For a more comprehensive understanding of the theoretical foundations discussed in this section – including radiation boundary conditions – the reader is referred to Ref. ^26^.

## Experiment

For the exploration of the properties of this finite photonic crystal, we focus on transmission spectra, as they are particularly suitable for comparing theoretical predictions with experimental results. The setup comprises a vector network analyser (model: N5245A from Agilent Technologies) and two logarithmic periodic broadband antennas (model: STLP 9149 from Schwarzbeck), connected via coaxial cables (model: elspec Duratest 150 Series from Teledyne Technologies). The antennas were mounted on tripods at a height of 1.06 meters, each positioned one meter from the outer stele row, with the antennas directed towards each other. Using the network analyzer, we measured the scattering parameters *S* for two distinct orientations, as indicated by the dashed lines in Fig. 2, both corresponding to the X (or Y) direction in reciprocal space. The horizontal line is denoted by $S^1$, and the vertical line by $S^2$. Data were recorded from sensor one to sensor two ($S_{21}$) and the reverse ($S_{12}$), while each curve is derived by averaging 10 individual measurements. The frequency response function is then defined as the mean of the different scattering parameters, as follows:

8 $$\begin{aligned} T(f) = \frac{1}{4}\Bigl(S_{12}^1 + S_{21}^1 + S_{12}^2 + S_{21}^2 \Bigr). \end{aligned}$$

The same definition is employed for the response spectrum, which was calculated using FEM.

## Results

The transmission characteristics of the periodic stele artwork, analyzed as a function of frequency, for both TM and TE modes, are shown in Fig. 3 and 4. Overall, the experimental measurements reveal pronounced frequency-dependent variations of the response function, aligning well with the simulated FEM spectra. This is consistent with the anticipated activity of a photonic crystal, as represented by the band structure. For both polarizations, we observe the occurrence of band gaps, within which waves cannot propagate due to the exclusive presence of evanescent solutions.

The band structure for TM modes, shows characteristic features such as directional Bragg gaps, opening at the high-symmetry points $\Gamma$ and X; they are evident as spectral dips in the response function. The measurements exhibit a strong agreement not only with the band structure but also with the simulated response. Notably, the cell of the latter closely resembles the experimental setup, as illustrated in Fig. 5 for the magnetic field.

For TE modes, the remarkable phenomenon of a cutoff band at 1 GHz, which can be interpreted as an effective plasma frequency of the periodic stelae system ^27^, leads to a significant omnidirectional response reduction at lower frequencies (as illustrated in Fig. 4). Around this band, a slight increase is recorded, which delineates the low-frequency band gap from the subsequent complete gap between 1 and 1.5 GHz. The increase in response is not much higher than in the gap regions, as coupling modes originating from the antenna with the flat-band modes within the artwork proves particularly challenging in the experiment – primarily due to the low group velocity. The second band is clearly indicated by a response maximum, followed by a dip that precisely corresponds to the third frequency gap.

Regarding the overall influence of defects, it was found that the different directions are indistinguishable in the simulated spectra. This finding is corroborated by the experiment, which reveals almost no difference between forward and backward measurements ($S_{12}$ or $S_{21}$) in the observed response.

In general, we can state that there is a consistent agreement between theory and experiment, especially when considering the numerous approximations and potential sources of error. These primarily include the following factors: the finiteness of the structure, the nine defects, the varying stele heights in the third dimension, and the fact that the frequency dependence of the permeability and permittivity function of the metal were not accounted for in the two-dimensional band structure calculations. Additionally, the scatterers are hollow and mounted on four interconnected metal ground plates. Due to the public nature of the measurement location, several external sources of influence were encountered, including: a $2\times 3~$ meter glass plate positioned adjacent to the setup, transient passers-by, bicycle parking stalls about four meters from the source, a parking area with vehicles approximately 200 meters behind. Furthermore, 5G equipment emitting a pulsed signal is installed on the roof of a neighboring building, at a considerable distance of approximately 150 meters – well outside the main radiation direction. The influence remains rather significant – despite the time-varying nature of the signal, no peaks could be observed on the network analyzer.

## Conclusions

With this article, we have demonstrated that art can convey additional meanings beyond the artist’s original intent. The sculpture by Stefan Nestler subjects radio waves to the principles of periodicity, which are confirmed by both photonic band structure and frequency response calculations that align well with the experiment. Our findings reveal that within the stele structure, there are frequency regions where electromagnetic wave propagation is confined to evanescent modes. With these results, we aim to draw attention to the potential of applying the concept of photonic crystals on scales much larger than those previously reported, thereby opening new avenues for specific applications in filtering, guiding, or localizing waves that fall within the telecommunications spectrum.

## Supplementary Information

Supplementary Information 1.

Supplementary Information 2.

Supplementary Information 3.

Supplementary Information 4.

Supplementary Information 5.

Supplementary Information 6.

## Data Availability

All numerical and experimentally obtained data are provided within the supplementary information files.
